# The role of *ESCO2*, *SALL4* and *TBX5* genes in the susceptibility to thalidomide teratogenesis

**DOI:** 10.1038/s41598-019-47739-8

**Published:** 2019-08-06

**Authors:** Julia do Amaral Gomes, Thayne Woycinck Kowalski, Lucas Rosa Fraga, Gabriel S. Macedo, Maria Teresa Vieira Sanseverino, Lavínia Schuler-Faccini, Fernanda Sales Luiz Vianna

**Affiliations:** 1 Programa de Pós-Graduação em Genética e Biologia Molecular, Universidade Federal do Rio Grande do Sul (UFRGS), Porto Alegre, Brazil; 2 Laboratório de Genética Médica e Evolução, Universidade Federal do Rio Grande do Sul (UFRGS), Porto Alegre, Brazil; 3 Instituto Nacional de Genética Médica Populacional (INAGEMP), Porto Alegre, Brazil; 4 Laboratório de Medicina Genômica, Hospital de Clínicas de Porto Alegre (HCPA), Porto Alegre, Brazil; 5 Sistema Nacional de Informações sobre Agentes Teratogênicos (SIAT), Serviço de Genética Médica, Hospital de Clínicas de Porto Alegre (HCPA), Porto Alegre, Brazil; 6 Departamento de Ciências Morfológicas, Instituto de Ciências Básicas da Saúde, Universidade Federal do Rio Grande do Sul (UFRGS), Porto Alegre, Brazil; 7 Laboratório de Pesquisa em Bioética e Ética na Ciência (LAPEBEC), Hospital de Clínicas de Porto Alegre (HCPA), Porto Alegre, Brazil

**Keywords:** Molecular biology, Genetics, Molecular biology, Genetics, Molecular biology

## Abstract

Thalidomide is widely used for several diseases; however, it causes malformations in embryos exposed during pregnancy. The complete understanding of the mechanisms by which thalidomide affects the embryo development has not yet been obtained. The phenotypic similarity makes TE a phenocopy of syndromes caused by mutations in *ESCO2*, *SALL4* and *TBX5* genes. Recently, SALL4 and TBX5 were demonstrated to be thalidomide targets. To understand if these genes act in the TE development, we sequenced them in 27 individuals with TE; we verified how thalidomide affect them in human pluripotent stem cells (hPSCs) through a differential gene expression (DGE) analysis from GSE63935; and we evaluated how these genes are functionally related through an interaction network analysis. We identified 8 variants in *ESCO2*, 15 in *SALL4* and 15 in *TBX5*. We compared allelic frequencies with data from ExAC, 1000 Genomes and ABraOM databases; eight variants were significantly different (p < 0.05). Eleven variants in *SALL4* and *TBX5* were previously associated with cardiac diseases or malformations; however, in TE sample there was no association. Variant effect prediction tools showed 97% of the variants with potential to influence in these genes regulation. DGE analysis showed a significant reduction of *ESCO2* in hPSCs after thalidomide exposure.

## Introduction

Thalidomide and its analogs – pomalidomide and lenalidomide - are drugs widely used worldwide for several conditions, such as erythema nodosum leprosum (ENL) - a skin condition related to Hansen’s disease (also known as leprosy) - and multiple myeloma^1^. These drugs have anti-inflammatory, immunomodulatory and antiangiogenic properties^2,3^; however, when used during pregnancy they cause Thalidomide Embryopathy (TE) in exposed embryos. From all these three drugs, only thalidomide is produced in Brazil^4–6^; the only country that still registers cases despite laws and regulations regarding its distribution^7^.

From epidemiological data collected in the 1960s, it is estimated that 20–50% of the embryos exposed to thalidomide present TE^8^. Thus, a large number do not develop an abnormal phenotype. The frequency of children born with a TE compatible phenotype has increased. From 1982 to 1999, in Brazilian hospitals, the frequency was around 1.92/10,000 births and from 2000 to 2008 around 3.10/10,000 births^9^. This phenomenon is probably explained by the higher availability of the drug for the treatment of ENL^9^. The molecular mechanisms of thalidomide teratogenicity are not fully elucidated, slowing down the development of a safe analog (i.e. without the teratogenic propriety). The embryonic genetic background is believed to act in the differential susceptibility to teratogen-induced damage^7,10–12^.

TE is considered a phenocopy of three genetic syndromes - Roberts syndrome, Duane-radial ray syndrome (also known as Okihiro syndrome) and Holt-Oram syndrome - since its features (caused by a non-genetic agent) resemble those phenotypes caused by mutations^13^ (Table 1). Roberts syndrome (RBS; MIM #268300), also known as Pseudothalidomide syndrome, is a rare autosomal recessive disorder caused by mutations in *ESCO2* gene. ESCO2 protein is an acetyltransferase responsible for cohesion of sister chromatids during cell division process^14,15^. Duane-radial ray syndrome (DRRS; MIM #607323) and Holt-Oram syndrome (HOS; MIM #142900) are both autosomal dominant conditions caused by mutations in *SALL4* and *TBX5* genes, respectively. These two genes encode transcription factors that interact and act on limb and heart development^16–19^.

**Table 1:** Similarities and differences - TE and genetic syndromes.

Characteristics/Phenocopies	Thalidomide Embriopathy	Roberts Syndrome	Duane Radial Ray Syndrome/Okihiro Syndrome	Holt-Oram Syndrome
Etiology	Teratogenic	Autosomal Recessive	Autosomal Dominant	Autosomal Dominant
Associated gene	Multifactorial	*ESCO2*	*SALL4*	*TBX5*
Molecular pathways to which these genes act	Angiogenesis; Oxidative stress; CUL4^CRBN^ complex	Cell Cycle; Establishment of Sister Chromatid Cohesion	Transcriptional regulation of pluripotent stem cells; Heart development and Limb development; Regulation of Wnt-mediated beta catenin signaling and target genes transcription	Heart development; Target genes transcription; Mesenchymal, Embryonic and pluripotent stem cell differentiation and lineage-specific markers; Human embryonic stem cell pluripotency; Limb development
Pre-axial limb reduction defect	Absent thumbs, duplicated or with three falanges, radius compromised without affecting the ulna	The thumb is often affected by proximal positioning or digitalization, hypoplasia or agenesis, long bones preferably affected in the following order: radius, ulna and humerus; fibulae, tibiae, and femur	Shortening and radial deviation of forearms, aplasia or hypoplasia of radius, bone hypoplasia of taenial region	Absence, hypoplasia or malformation of tendon bones, carpi and radius
Facial	Cleft lips and/or palate (rare), midline hemangioma	Cleft lips and/or palate, microcephaly, hypertelorism	Hypertelorism	Not reported
Ocular	Microphthalmia, coloboma	Prominent eyes, corneal opacification	Duane unilateral or bilateral anomaly	They are not characteristic
Auditory	Anotia, microtia, deafness	Hypoplastic lobes	Conductive or sensorineural deafness	They are not characteristic
Cardiac	Ventricular septal defect, aortic coagulation, tetralogy of Fallot	Ostium secundum atrial septal defect, ventricular septal defect, cardiac conduction defects	Ventricular septal defect, atrial septal defect, tetralogy of Fallot	Atrial septal defect, arrhythmias, ventricular septal defect
Genitalia	Cryptorchidism, bicorn or hypoplastic uterus, small or absent penis	Cryptorchidism, large penis relative to the body, increased clitoris	Not reported	Not reported

References^3,7,18,31,43–49^.

Some syndromes are caused by mutations in genes that encode proteins that are affected by some teratogens. Thus, the analysis of the molecular bases of some syndromes has been efficient in the understanding of teratogenic mechanisms^20^. *TBX5* and *SALL4* gene expression is reduced in wing buds of chicken embryos and primary human embryonic fibroblasts exposed to thalidomide^21^. TBX5 was recently demonstrated to be a direct target of thalidomide; in the presence of the drug, the protein dramatically reduces its DNA binding potential and the activation of its target genes. The drug also prevents TBX5 binding to HAND2, an important protein involved in the heart development^22^. Even more recently, two studies suggested a new hypothesis to thalidomide teratogenesis, highlighting the SALL4 protein degradation as the mechanism responsible for the malformations observed in TE. These studies demonstrated that SALL4 is degraded post-transcritionally in rabbits and different types of human cell lines after thalidomide or its analogs exposure, but not in models not sensitive to thalidomide, such as mice^23,24^.

Taking into account the phenotypic similarity between people with TE and individuals with the aforementioned genetic syndromes, and also the experimental studies showing that these genes are affected by thalidomide, the investigation of these genes might explain at least partially why some people are affected by TE and others are not. Thus, in order to evaluate the role of *ESCO2*, *TBX5* and *SALL4* genes in thalidomide teratogenesis we have sequenced these genes in individuals with TE and analyzed the variants found in relation to their potential to increase the risk to TE development. After that, we verified - from transcriptomes available in the Gene Expression Omnibus (GEO) database - how thalidomide affects their expression on human pluripotent stem-cells (hPSC) exposed to the drug. Finally, we verified - through systems biology databases - if and how these genes interact with each other.

## Results

### Gene panel sequencing of individuals with Thalidomide Embryopathy and *in silico* functional predictions

Twenty-seven Brazilian subjects with TE were included in our study. The characterization of these individuals, regarding their congenital anomalies and late outset diseases, is described in Table 2.

**Table 2:** Clinical characterization of individuals with Thalidomide Embryopathy.

Characteristics	Number of individuals	%
*Male*	17/27	63
**Congenital Anomalies** ^**a**^		
*Upper limbs*	26/27	96
*Lower limbs*	10/27	37
*Ears*	3/17	18
*Eyes*	6/18	33
*Heart*	1/17	6
*Genital tract*	2/17	12
*Skeletal*	4/12	33
**Late Diseases** ^**a**^		
*Hearing deficiency*	7/16	44
*Visual deficiency*	12/15	80
*Cardiovascular disease*	4/16	25
*Psychological disturbance*	6/16	38

^a^Data not available for all 27 individuals of the TE sample.

**Figure 1:**
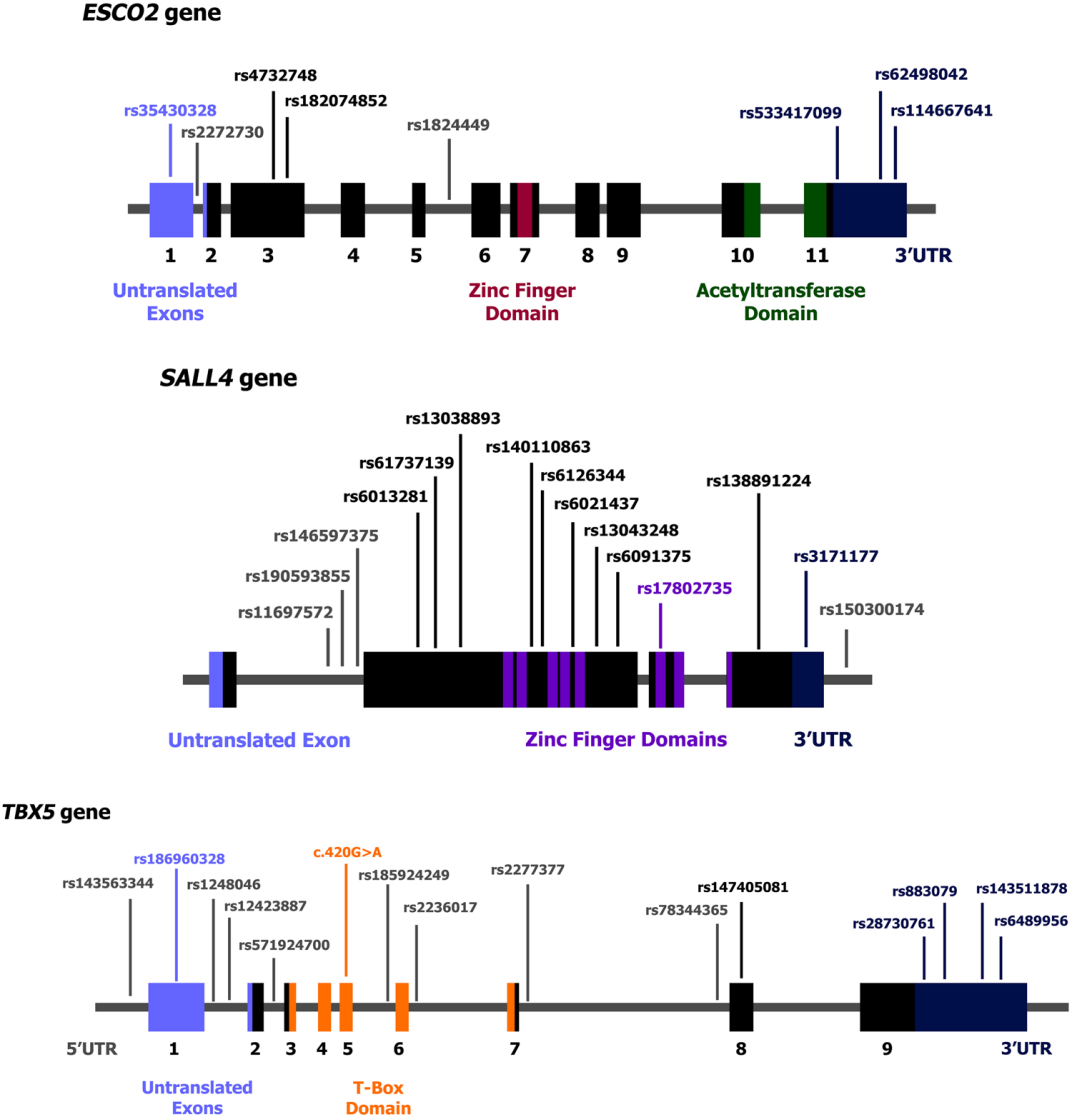
Position of variants found in exons, flanking introns and untranslated regions of *ESCO2*, *SALL4* and *TBX5* genes in people with Thalidomide Embryopathy. We identified 8 variants in *ESCO2* gene, 15 in *SALL4* gene and 15 in *TBX5* gene (one of them – c.420 C > T (p. Asp140=) – a novel variant).

Using a targeted sequencing approach, we sequenced exons, flanking intronic regions, and untranslated regions of *ESCO2*, *SALL4* and *TBX5* genes. The average depth of coverage for the variants found was 491.5×. A coverage higher than 100x was obtained in 92.44% of the bases. Gene sequencing data has been deposited at the Sequence Read Archive (SRA) under accession number SRP160424. A novel variant has been submitted to Leiden Open Variation Database (LOVD). Figure 1 and Table 3 summarize all variants found and their position in each gene.

**Table 3:** Allelic frequencies of the variants found in the individuals with Thalidome Embryopathy compared to the ones of different databases.

Gene	Polymorphism	DNA variation	Aminoacid variation	Position	MAF in the TE	MAF in the ExAC	*p* ^a^	MAF in the 1000 Genomes	*p* ^a^	MAF in the ABraOM	*p* ^a^
*ESCO2*	rs35430328	c.-151G > A		Exon 1 (UTR)	0.259			0.354 (A)	0.532	0.273	0.986
	rs2272730	c.-17 + 19 C > T		Intron	0.463			0.424 (T)	0.938	0.379	0.742
	rs4732748	c.239 C > T	p.Ala80Val	Exon 3	0.056	0.099	0.736	0.107 (T)	0.655	0.102	0.747
	rs182074852	c.346 G > A	p.Asp116Asn	Exon 3	0.019			<0.001 (A)	0.209		
	rs1824449	c.1013 + 35 G > A		Intron	0.111	<0.001	<0.003	<0.001 (G)	<0.018	0.015	<0.013
	rs533417099	c.*71_*74delTATT		3′UTR	0.056			<0.001 (-)	<0.018	0.002	0.013
	rs62498042	c.*130 G > A		3′UTR	0.241			0.458 (G)	0.020	0.244	1.000
	rs114667641	c.*1489 A > T		3′UTR	0.019			<0.001 (T)	0.209	0.045	0.760
*SALL4*	rs11697572	c.131–316 C > T		Intron	0.056			0.037 (T)	0.696		
	rs190593855	c.131–260 G > A		Intron	0.019			0.007 (A)	0.655		
	rs146597375	c.131–226 T > C		Intron	0.019			0.017 (C)	0.782		
	rs6013281	c.540 T > C	p.Asn180=	Exon 2	0.019	0.005	0.847	0.004 (T)	0.567	0.002	0.470
	rs61737139	c.645 C > G	p.Leu215=	Exon 2	0.037	0.025	0.969	0.022 (C)	0.655	0.020	0.743
	rs13038893	c.1056 G > A	p.Ala352=	Exon 2	0.333	0.313	0.969	0.328 (A)	1.000	0.266	0.742
	rs140110863	c.1353 C > T	p.Ala451=	Exon 2	0.019	<0.001	<0.003	<0.001 (T)	0.209		
	rs6126344	c.1520 T > G	p.Leu507Arg	Exon 2	0.315	0.350	0.474	0.335 (G)	0.938	0.350	0.813
	rs6021437	c.1860 A > G	p.Thr620=	Exon 2	0.315	0.350	0.847	0.336 (G)	0.938	0.362	0.760
	rs13043248	c.2037 C > T	p.Thr679=	Exon 2	0.148	0.102	0.736	0.115 (T)	0.757	0.069	0.351
	rs6091375	c.2392 A > C	p.Ile798Leu	Exon 2	0.074	0.052	0.847	0.065 (C)	0.898	0.073	1.000
	rs17802735	c.2640 G > C	p.Ser880=	Exon 3	0.093	0.089	1.000	0.127 (G)	0.828	0.083	0.939
	rs138891224	c.2977 G > C	p.Gly993Arg	Exon 4	0.019	<0.001	<0.003	<0.001 (C)	0.049		
	rs3171177	c.*125 T > A		3′UTR	0.019			0.041 (T)	1.000	0.057	0.747
	rs150300174	c.*497 T > C		Downstream	0.019			0.004 (C)	0.567		
*TBX5*	rs143563344	c.-712 C > T		5′UTR	0.019			0.014 (T)	0.214		
	rs186960328	c.-664 G > A		Exon 1 (UTR)	0.037			<0.001 (A)	0.777		
	rs1248046	c.-39 + 113 A > G		Intron	0.398			0.416 (G)	0.898	0.339	0.760
	rs12423887	c.-38–1865 G > A		Intron	0.167			0.130 (A)	0.662	0.123	0.760
	rs571924700	c.147 + 107 C > A		Intron	0.037			<0.001 (A)	0.022		
	Novel variant	c.420 C > T	p. Asp140=	Exon 5	0.019						
	rs185924249	c.511–56 T > C		Intron	0.019			0.008 (C)	0.655	<0.001	0.448
	rs2236017	c.663 + 36 G > T		Intron	0.352	0.390	0.847	0.395 (G)	0.781	0.403	0.760
	rs2277377	c.755 + 94 C > A		Intron	0.241			0.307 (A)	0.655	0.251	1.000
	rs78344365	c.756–26 G > A		Intron	0.019	<0.001	<0.003	0.001 (A)	0.333	0.004	0.742
	rs147405081	c.787 G > A	p.Val263Met	Exon 8	0.037	<0.001	<0.003	<0.001 (A)	0.022	0.002	0.090
	rs28730761	c.*77 A > G		3′UTR	0.074	0.169	0.225	0.123 (G)	0.655	0.100	0.813
	rs883079	c.*97 G > A		3′UTR	0.389	0.258	0.113	0.285 (G)	0.379	0.349	0.760
	rs143511878	c.*812 C > T		3′UTR	0.019			0.008 (T)	0.655	0.012	0.760
	rs6489956	c.*1101 A > G		3′UTR	0.222			0.190 (A)	0.782	0.333	0.463

Reference sequences are: *ESCO2* transcript NM_001017420, *SALL4* transcript NM_020436 and *TBX5* transcript NM_000192. ^a^With FDR correction.

The frequency of all the variants was in accordance with the Hardy-Weinberg equilibrium, except for c.1013 + 35 G > A and c.*71_*74delTATT of *ESCO2* gene. We observed differences in the allelic frequencies of eight variants between the TE group and the genomic databases (ExAC, 1000 Genomes and ABraOM) (Table 3), three in *ESCO2*, two in *SALL4* and three in *TBX5*. Three of them (from *SALL4* and *TBX5* genes) were in coding sequences.

There was no association between the twelve rare variants identified (frequency < 0.01 in all databases) and the presence of some specific congenital anomaly or disease observed in the TE group.

Variants in the same gene demonstrated high linkage disequilibrium (D’ > 0.9 and LOD > 2) (Supplementary Table S2). The haplotypes inferred for each gene (Supplementary Table S3) were also not associated with specific congenital anomalies or diseases in the TE group.

**Figure 2:**
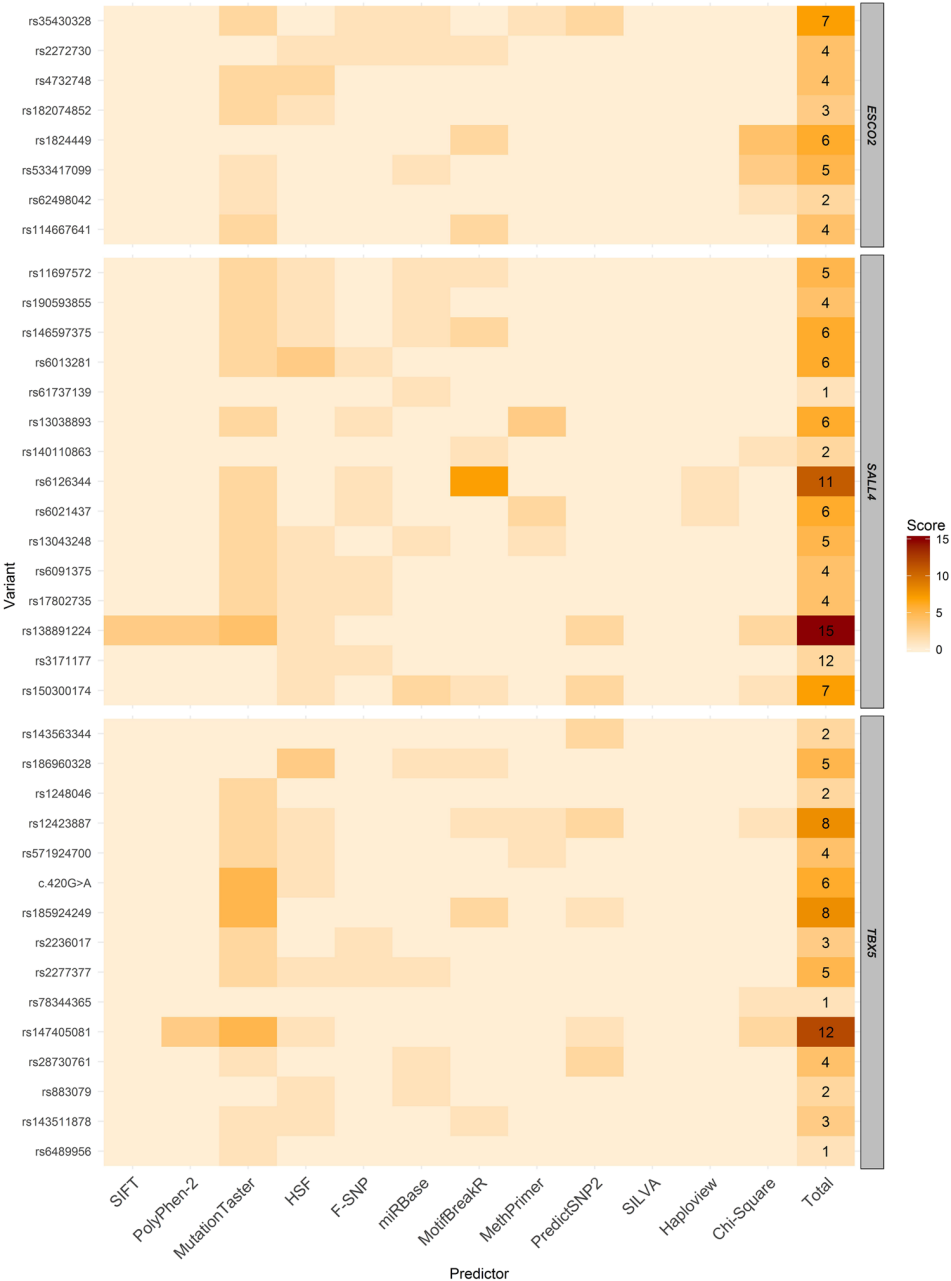
Heatmap representing the potential impact of variants found in *ESCO2*, *SALL4* and *TBX5* genes in regulatory features of these genes, their proteins and in TE. In the lines are represented the variants, the scores assigned to them after functional predictions and the final score. The scores are presented by color tone variation; the higher the points the darker the color; the lower the points the lighter the color. Regarding the columns, the first ten represent variant effect prediction tools, the eleventh represents the formation of haplotypic blocks and the twelfth represents the statistically significant difference of some variant with the genomic databases. The variant effect prediction tools pointed out variants which altered the features evaluated by the tool, being the impact of lesser or greater degree. To haplotypic blocks, it received a point variants that formed block. Variants with a statistically significant difference of allelic frequencies between the TE sample and the databases were pointed out, being greater the score of variants which differed from more than one database.

According to the functional prediction tools, some variants potentially affect different aspects of the gene or the protein (Fig. 2) (Supplementary Table S4). They were also more frequent than expected in the TE group. Thus, they stood out, being more likely to influence in the TE susceptibility and in the risk of development of specific congenital anomalies identified in the affected individuals (Fig. 2) (Supplementary Tables S4 and S5).

#### *ESCO2* gene revealed few variants, highlighting the regulatory ones

Eight variants were found in *ESCO2* gene in all individuals (Fig. 1 and Table 3). Three variants located in regulatory regions stood out according to a score we developed (based in the variants effect according to prediction tools and their frequencies in the TE group): c.−151 G > A, c.1013 + 35 G > A and c.*71_*74delTATT (Fig. 2).

The 5′UTR c.−151 G > A was predicted as pathogenic, affecting the splicing, one CpG island and one miRNA binding site (hsa-miR-6858-3p) (Supplementary Table S4). The intronic variant c.1013 + 35 G > A, reported as benign in the ClinVar database^25^, was significantly more prevalent in the TE group than in the evaluated databases (Table 3) and was predicted to affect two transcription factors binding sites (TBP, which has a binding site in *ESCO2* and POU6F1, which acts in heart development)^25^ (Supplementary Table S4). The 3′UTR c.*71_*74delTATT was also significantly more frequent in TE group (Table 3) and it affects splicing and two miRNAs binding sites (hsa-miR-606 and hsa-miR-3149) (Supplementary Table S4).

#### *SALL4* gene presented many variants in coding regions with potential to affect splicing and transcription factors binding sites

We identified 15 variants in *SALL4* gene, 10 of them in coding regions (Fig. 1 and Table 3). Five variants stood out by our score: c.2977 G > C (p.Gly993Arg), c.1520 T > G, c.*497 T > C, c.1860 A > G (p.Thr620=) and c.131-226 T > C (Fig. 2).

The missense c.2977 G > C (p.Gly993Arg) and the downstream c.*497 T > C were significantly more frequent in the TE group (Table 3); they were predicted as pathogenic and affecting splicing; the c.*497 T > C also affecting three miRNAs (hsa-miR-5095, hsa-miR-1254 and hsa-miR-5689) and one transcription factor binding site (PITX2, which acts in heart and limb development)^25^ (Supplementary Table S4). The missense c.1520 T > G (p.Leu507Arg) and the synonymous c.1860 A > G (p.Thr620=) were in a haplotype block and were predicted as affecting splicing; the first disrupts seven transcription factors binding sites (PAX5, PPARG and RXRB, which have binding sites in *SALL4* and PPARA, PPARD, RARB and RXRA, that act in heart development)^25^; the last disrupts two CpG islands (Supplementary Table S4). The intronic c.131–226 T > C possibly affects the splicing, one miRNA (hsa-miR-615-5p) and two transcription factors binding sites (TFAP4 and ZBTB7B, which regulate in *SALL4*)^25^ (Supplementary Table S4).

#### In *TBX5* a novel variant within the T-box domain was identified and many variants were predicted as pathogenic

We identified 15 variants in *TBX5* gene, one of them – NM_000192:c.420 C > T (p. Asp140=) – has not been reported in genomic databases (Fig. 1 and Table 3). This novel variant was identified in heterozygosis in one individual, with a coverage of 497x in the sequencing (50% for each nucleotide). Four variants of *TBX5* stood out by our score: c.787 G > A (p.Val263Met), c.−38-1865 G > A, c.511-56 T > C and the novel c.420 C > T (p. Asp140=) (Fig. 2).

The missense c.787 G > A (p.Val263Met), which was not located in the T-box domain, was significantly more frequent in the TE group (Table 3) and predicted as pathogenic and affecting splicing (Supplementary Table S4). In the TE group, two individuals had this variant; one of them presents angina (a chest pain due to reduced blood flow to the heart). The intronic variants c.-38-1865 G > A and c.511-56 T > C were predicted as pathogenic and affecting splicing. The first affects also one CpG island and one transcription factor binding site (NFIB, that has a binding site in *TBX5*)^25^. The second affects two transcription factors binding sites (GATA1 and GATA2, which act in heart development)^25^ (Supplementary Table S4).

A novel synonymous variant c.420 C > T (p. Asp140=) was located within the T-Box domain region of the gene. It was predicted as pathogenic and affecting splicing (Supplementary Table S4). Once it was never described, we classified it based on the classification criteria of the American College of Medical Genetics and Genomics/Association for Molecular Pathology (ACMG/AMP) Standards and Guidelines^26^. The included criteria were: (1) Absent from controls (or at least low frequency if recessive) in Exome Sequencing Project, 1000 Genomes Project, or Exome Aggregation Consortium and (2) Multiple lines of computational evidence support a deleterious effect on the gene or gene product (conservation, evolutionary, splicing impact, etc.). According ACMG/AMP system this alteration was classified as a variant of uncertain significance (VUS).

### Differential Gene Expression (DGE) analysis from secondary data of the GEO database

The expression of *ESCO2*, *SALL4* and *TBX5* genes in human pluripotent stem-cells after thalidomide exposure was evaluated from secondary data obtained in the GSE63935 study, available in the GEO database^27^. The differential gene expression (DGE) analyses demonstrated a sharp reduction in *ESCO2* expression after 2 and 6 days of thalidomide exposure (p = 1.39e-09 and 0.045699) (Table 4).

**Table 4:** Effects of thalidomide exposure on *ESCO2*, *TBX5*, *SALL4* gene expression in human pluripotent stem-cells, compared to saline solution exposure.

	LogFC (2 days)	Adj P-Value	LogFC (6 days)	Adj P-Value
*ESCO2*	−2.08935	1.39e-09	−1.30927	0.045699
*TBX5*	−1.17209	0.492226	−2.86675	0.154217
*SALL4*	−0.44506	0.294376	−0.95945	0.083761

LogFC: Logarithmic Fold Change.

### Interaction networks and gene ontology analysis

**Figure 3:**
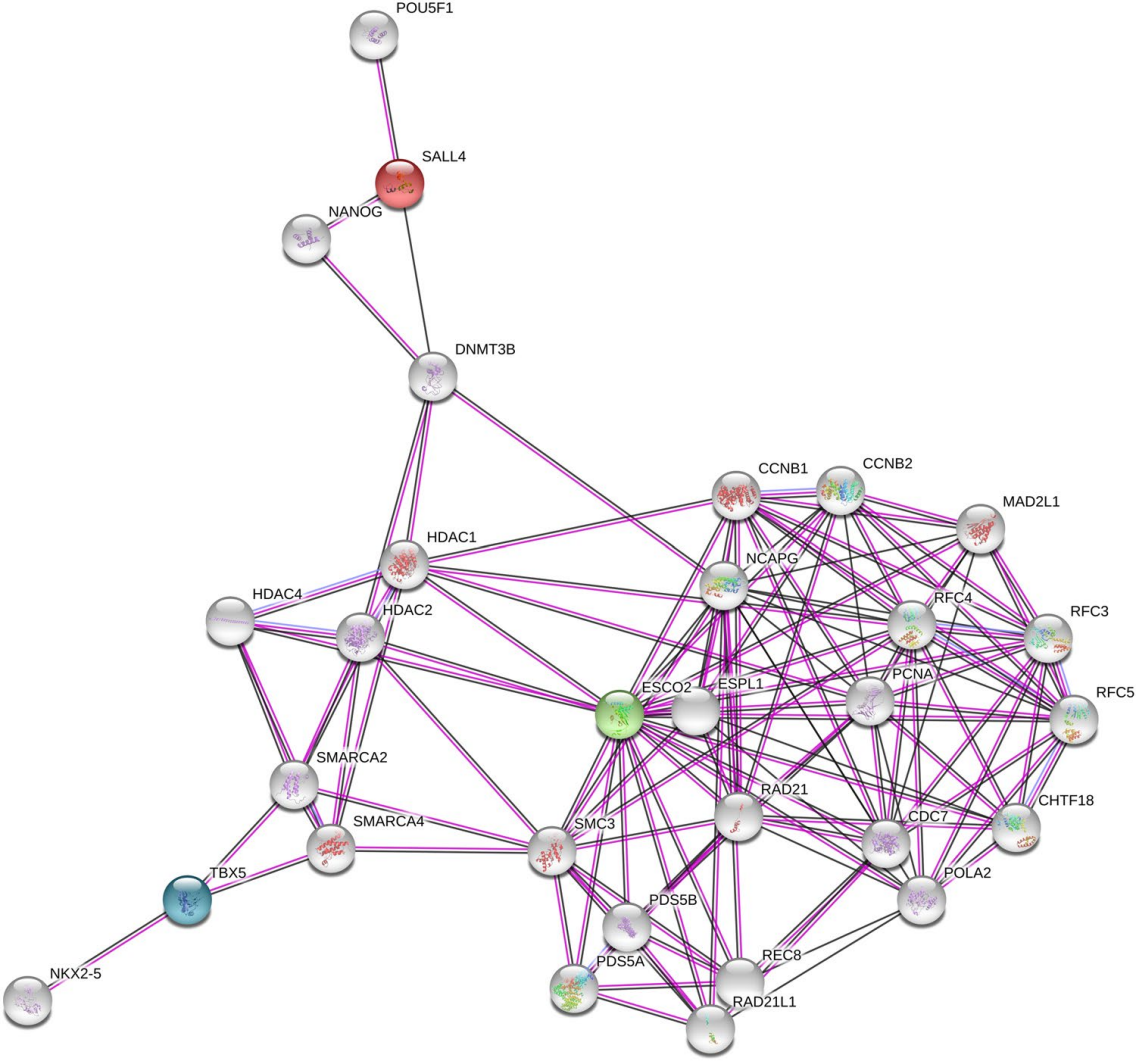
Protein–protein interaction network including ESCO2, SALL4 and TBX5 proteins. These three proteins do not interact directly, but through others secondary binding targets.

The interaction network analyses performed in STRING database showed that ESCO2, SALL4 and TBX5 proteins do not interact directly, although they are included in the same network through interactions with other proteins (Fig. 3). A Gene Ontology (GO) analysis identified 160 biological processes significantly enriched these genes’ network, mainly linked to cell cycle and DNA replication (Supplementary Table S6). SALL4 and TBX5 share, as expected, ontologies of embryonic, limb and heart development.

## Discussion

Although the thalidomide teratogenic effect has been known for almost 60 years, new cases of TE are still reported in Brazil^7^. Studies have attempted to understand the mechanisms by which thalidomide affects the embryo development; however, the complete understanding has not yet been obtained. The discovery of genes or proteins that are affected by thalidomide or somehow influence the susceptibility of the thalidomide teratogenic action is extremely important for the better understanding of the teratogenic mechanisms of thalidomide.

In this study we investigated a new hypothesis regarding thalidomide teratogenesis. The identification of teratogenic mechanisms is extremely difficult since teratogenesis is a multifactorial process^10^. Some teratogenic mechanisms could be clarified through the analysis of the molecular bases of genetic disorders phenocopies of embryopathies due to drugs exposure^20^. Here we evaluated three genes that are the genetic basis of syndromes in which TE is a phenocopy.

The relationship of thalidomide with SALL4 and TBX5 proteins was recently demonstrated by three experimental studies^22–24^. Thalidomide is capable of binding to TBX5 protein^22^ and it is able to induce SALL4 degradation^23,24^. Both studies suggested that such capacities of thalidomide in SALL4 and TBX5 proteins could be the possible mechanisms by which thalidomide causes TE. In this way, our approach became even more relevant, providing new insights about the role of these genes in the genetic susceptibility to TE in humans.

In *ESCO2* gene, the regulatory variants that we found showed to have the greatest pathogenic potential to increase the risk to TE. It is known that regulatory variants could increase the susceptibility to diseases since they can act in transcription, splicing and translation processes^28^; however, we did not find an association between their frequencies in the TE group and an increased risk for this condition.

Regarding the *SALL4* gene, some variants were highlighted to their potential risk for TE or specific anomalies. The rare missense c.2977 G > C (p.Gly993Arg), predicted as pathogenic and affecting splicing, was present only in one individual in the TE group. Because of the absence of pathogenicity information for this variant in the literature and the scarcity of clinical information from this carrier, we did not consider it as causative of an anomaly or syndrome on its carrier. Two variants in coding regions – c.1520 T > G (p.Leu507Arg) and c.1860 A > G (pThr620=) – were in a haplotype block. Haplotypes have been already associated with a risk for teratogenesis^29^, including thalidomide teratogenesis^11^; however, despite some of these variants carriers present auditory defect (4/14 carriers), visual impairment (6/14) and cardiovascular diseases (3/14), these anomalies could be not associated with the frequency of this haplotype block. Finally, the 3′UTR c.*497 T > C appeared affecting the binding the miRNA hsa-miR-1254. This miRNA is expressed in human embryonic stem cells and it regulates genes of stem cells differentiation, development and transcriptional regulation^30^, compatible with *SALL4* function. The carriers of this variant did not have any exclusive congenital anomaly that could be associated with its presence.

SALL4 protein was recently demonstrated to be degraded post-transcriptionally after thalidomide or its analogs exposure in different types of human cells^23,24^. SALL4 degradation is mediate through thalidomide-dependent Cereblon protein (CRBN)^23,24^, an integrant of the CRL4^CRBN^ ubiquitin-ligase complex, previously described as the primary target of thalidomide^31^. The residue G416 in the second zinc finger domain of SALL4 was essential for its recruitment and degradation; also, the residue G600 of the fourth zinc finger domain had its importance highlighted^23,24^. Here, we did not identify any variation in these reported positions in the TE group. Similarly, there are no variants reported in humans for the residue G416 of *SALL4* in the genomic databases (ExAC, 1000 Genomes and AbraOM). For the residue G600, just one variant – NM_020436:c.1798 G > A (p.Gly600Ser) - was identified (frequency < 0.003) in these databases. Thus, comparing our findings in the TE group and data from genomic databases with the studies aforementioned, it is not possible to explain based on the *SALL4* variants why only 20–50% of the individuals exposed to thalidomide developed TE^8^.

Cardiac abnormalities are frequent in individuals with TE, with an estimated frequency of 8%^32^. *SALL4* and *TBX5* genes are essential to heart development^19,33^ and changes in them have been already associated to increased risk for cardiac diseases^34–39^. Here, eleven variants found in *SALL4* and *TBX5* were previously evaluated or associated with cardiac malformations or cardiovascular diseases (Supplementary Table S7)^34,36–38^. It is possible that the small sample size of this study did not allow us to find an association of these variants with increased risk for malformations or cardiovascular diseases in individuals with TE; however, it is quite likely that at least some of the variants found in these genes may play an important role in such conditions in these individuals, being important a further genetic and transcriptional evaluation of them in larger samples and also in experimental assays.

TBX5 action in heart development was demonstrated to be affected by thalidomide due to the drug impaired TBX5 and HAND2 proteins interaction^22^. Here, we did not identify in our TE sample any variant reported as important for TBX5 binding to DNA or to HAND2, which could be associated with cardiac malformations or related diseases. We found a missense variant c.787 G > A (p.Val263Met) in these gene in two individuals of the TE group. This variant was previously associated with bicuspid aortic valve^37^. One of the carriers has angina, but we cannot associate this phenotype with its presence since we observed it just in on individual.

Although being in the T-Box domain and having been predicted as pathogenic, the novel synonymous c.420 C > T (p. Asp140=) of *TBX5* was classified as VUS using the ACMG criteria. Because of that, we have not considered it as causing Holt-Oram Syndrome in the carrier. Moreover, the carrier does not have any exclusive anomaly that could to be caused by this variant.

Changes in *SALL4* and *TBX5* genes expression and SALL4 and TBX5 proteins levels after thalidomide exposure have been demonstrated^21,23,24^; however, *ESCO2* differential expression was not reported^23^. Here, we observed thalidomide drastically reducing *ESCO2* expression in human pluripotent stem-cells. Because ESCO2 protein plays a key role during development, its reduction in cells directly affect the development, causing malformations such as those seen in Roberts Syndrome^15^. Taking this into account, it is possible to suggest that the effect of thalidomide on this gene expression helps in the TE development, and this would also explain the mechanism by which TE is a phenocopy of Roberts Syndrome.

The sample size of this work is a limitation of our investigation. This sample was recruited through a collaboration with the Brazilian Association of Patients with Thalidomide Syndrome (ABPST) and represents more than 10% of the live people with Thalidomide Embryopathy (TE) in Brazil. However, there is great difficulty to expand this sample, because the majority of the individuals with TE are elderly and live in different regions in Brazil, not favoring recruitments and not attending ABPST meetings. Taking into account that new cases are not expected due to birth control in women taking thalidomide, this in the only Brazilian resource of recruitment. The evaluation of a control group, including people who were exposed to thalidomide during pregnancy but did not develop TE, would be the best epidemiological design for comparison with the cases of our sample. However, the information about which mothers used thalidomide during pregnancy and had children without TE in 1960’s is very difficult to recover. Many of these mothers are already deceased, others are in advanced age and do not remember such information. Epidemic TE cases occurred more than 50 years ago, and even if we tried to include a control sample from that time, it would probably be a biased information due to the time elapsed since then. In order to provide a genetic background for comparison of the frequency of variants, we have used public genome databases from different ethnic groups, including healthy Brazilians. Nevertheless, this is the only sample of survivors of thalidomide embryopathy which were born in 1960´s that has been widely assessed regarding genomic and clinical features. This approach can provide different and valuable insights about mechanisms and pathways of thalidomide involved in embryology and therapeutic actions in humans. Another limiting factor is the expression of the genes investigated in adults. Previous studies show that *SALL4* and *TBX5* have a high expression during development and then decrease their expression and restrict it to only a few tissues^40,41^. For this reason, we decided to evaluate the effect of thalidomide on the expression of *SALL4*, *TBX5* and *ESCO2* in cells representing the stage of embryonic development. Finally, few studies in literature evaluated the sequence of these three genes in humans, increasing the difficulty to compare and make hypotheses about genetic variability and its impact on the proteins.

Here we investigated a different hypothesis of those traditionally studied in thalidomide teratogenesis, evaluating the role of the genes that cause syndromes whose TE is a phenocopy. From the sequencing data of this study, we cannot conclude that the genetic variants found in *ESCO2*, *SALL4* and *TBX5* in our sample of people with TE could act in the genetic susceptibility to the TE development; however, this approach showed that variants in *SALL4* and *TBX5* with a known impact on cardiac malformations or diseases are not uncommon in individuals with TE. We could also demonstrate that genetic variants recently described as associated with thalidomide-TBX5 binding and SALL4 degradation thalidomide-mediate^22–24^ - proposed as responsible for the thalidomide teratogenesis - do not appear in our TE group. Moreover, we described a novel synonymous variant in the T-Box domain of *TBX5* gene, the most important domain of this transcription factor, and we classified it as VUS, being necessary its experimental validation. From the analysis of secondary gene expression data, which included a group of human pluripotent stem-cells exposed to thalidomide (cases) and an unexposed group (control), we have described for the first time that exposure to thalidomide is capable to affect *ESCO2* gene expression, a gene essential for cell division. Such result points to the *ESCO2* gene as a possible target for thalidomide, primary or secondary, that should be taken into account in future studies on thalidomide teratogenesis mechanisms as well as in investigations on thalidomide safe analogues.

Phenotypic evaluations are an important alternative to provide insights in molecular genetics researches. Regarding the current research, it is clear that further studies are necessary, although we demonstrated the importance of understanding how thalidomide mimics genetic syndromes to also comprehend its teratogenic property.

## Materials and Methods

### Ethical considerations

This study was approved by the Research Ethics Committee of the Hospital de Clínicas de Porto Alegre (number 10-0244). The whole research was performed in accordance with relevant guidelines and regulations. All subjects assigned a free and informed consent form.

### Sample

The 27 subjects of our sample were recruited through the Brazilian Association of People Affected by Thalidomide Syndrome (ABPST). This sample represents about 10% of the live cases of TE in Brazil.

### Molecular analysis

Saliva was collected through Oragene-DNA OG-500 (DNA Genotek, Canada) and DNA was obtained according to the manufacturer’s instructions.

A gene panel including *ESCO2*, *SALL4* and *TBX5* genes was designed through Ion Ampliseq^TM^ Designer tool (Thermo Fisher Scientific, USA) covering the coding regions and 50 bp of adjacent introns of each gene. The targeted gene sequencing was performed in Ion PGM technology (Thermo Fisher Scientific, USA) at Hospital de Clínicas de Porto Alegre. The sequences obtained were analyzed through Ion Reporter v.5.2 (Thermo Fisher Scientific, USA) using the genome reference GRCh37. The sequences of reference transcripts used were: NM_001017420 to *ESCO2*, NM_020436 to *SALL4* and NM_000192 to *TBX5*.

### Statistical analysis

We compared the allelic frequencies found in the TE sample with data from three different databases: 1000 Genomes Project (European population), Exome Aggregation Consortium (ExAC) (Europeans non-finnish) and Arquivo Brasileiro Online de Mutações (ABraOM) (Brazilian population database) using Chi-square or Fisher’s Exact Test in SPSS® v.18 (SPSS, IBM, USA) software. The FDR correction was used to eliminate the false discover rate. A two-tailed p-value < 0.05 was considered significant.

Linkage disequilibrium between variants was estimated using the Haploview program v.4.2 (IBM, USA), and the haplotypes were obtained with Bayesian algorithm in the PHASE v.2.1.1 tool (University of Chicago, USA).

### *In silico* analyses

*In silico* analyses of the variants found were performed with the following bioinformatics tools: SIFT, PolyPhen-2, F-SNP, Mutation Taster, Predict SNP-2, SILVA, MotifbreakR, HSF, MethPrimer and miRBase. Characteristcs of each tool used is described in Supplementary Table S1.

### Pathogenicity score

In order to evaluate the pathogenic potential of the variants found, which could increase the susceptibility to TE, we set up a score for each variant based in the statistics analyses (difference of allelic frequencies between TE group and genomic databases), haplotype block formation and *in silico* predictions of pathogenicity. If the variant affected some of these features it was scored and at the end we added the points.

### Differential gene expression (DGE) analysis

The raw data of the transcriptome GSE63935 available on the Gene Expression Omnibus (GEO) database^27^ was reassessed here. In the aforementioned study the RNA of human pluripotent stem cell-derived neural constructs (mix of neural, endothelial, mesenchymal and macrophage precursor) was extracted 2 and 6 days after exposure to toxicants. Here we compared the expression data of *ESCO2*, *SALL4* and *TBX5* genes in both periods after thalidomide exposure and compared with control cells (exposed to saline solution). Secondary data analysis was performed through edgeR package in RStudio v.1.0.136. P-values were adjusted by false discovery rate correction, considering an Adjusted P-value of <0.05 significant.

### Interaction networks and gene ontology analysis

We performed protein-protein interaction network analysis and gene ontology enrichment analysis in the STRING database^42^.

## Supplementary information

Supplementary Information

## Data Availability

The datasets generated during and/or analyzed during the current study are available from the corresponding author on reasonable request.
