# Supplementary material for: The role of ESCO2, SALL4 and TBX5 genes in the susceptibility to thalidomide teratogenesis
Source: Sci Rep. 2019 Aug 6;9:11413. doi: 10.1038/s41598-019-47739-8 (PMC6684595; doi:10.1038/s41598-019-47739-8)
Supplement: Supplementary file 1 — Supplementary Information [file 41598_2019_47739_MOESM1_ESM.pdf]

The role of ESCO2, SALL4 and TBX5 genes in the susceptibility to thalidomide teratogenesis

Julia do Amaral Gomes, Thayne Woycinck Kowalski, Lucas Rosa Fraga, Gabriel de Souza Macedo, Maria Teresa Vieira Sanseverino, Lavínia Schuler-Faccini and Fernanda Sales Luiz Vianna

Supplementary Table 1. Bioinformatic tools used in the *in silico* analyses and theirs respective functions.

Predictor	Function	Reference
(1) Sorting Intolerant From Tolerant (SIFT)	Compiles a dataset of functionally related protein sequences and uses a sequence homology-based approach to classify aminoacid substitution	Kumar et al., 2009
(2) Polymorphism Phenotyping v.2 (PolyPhen-2)	Uses sequence-based and structure-based predictive features to predicts the impact of aminoacid changes in a human protein	Adzhubei et al., 2010
(3) Functional Single Nucleotide Polymorphism (F-SNP)	Compiles functional information about SNPs from 16 bioinformatics tools and databases, and predicts both functional effects in protein as disruption of regions known to be splice sites and transcriptional regulation sites	Lee and Shatkay, 2008
(4) Mutation Taster	Evaluates the difference between the physico-chemical characteristics of original versus new amino acids to predict functional effects, and evaluates only donor and acceptor splice sequences to predict possible splicing changes	Site: http://www.mutationtaster.org/in fo /documentation.html
(5) Predict SNP - 2	Includes 6 tools to predict theimpact of the variants: 1) DANN, a deep neural network-based classifier that capture non-linear relationships among features; 2) FATHMM-MKL, that assesses the functional impact using an SVM model; 3) GWAVA, which is based on a random forest classifier and is designed for the analysis of regulatory variants; 4) FunSeq2, that uses an empirical scoring system that integrates evolutionary constraints, epigenetic data and knowledge of transcription-binding motifs to assess the impact of variants; 5) FitCons, that defines clusters of similar functional genomic signals and estimates the functional impact of variants with the same fingerprint on the basis of allele frequency distributions in human populations; 6) CADD, which based on a logistic regression model, takes into account evolutionary conservation, regulatory and transcript information, and protein-level scores.	Bendl et al., 2016
(6) SILVA	A tool for the automated harmfulness prediction of synonymous (silent) mutations within the human genome. SilVA bases its predictions on a number of features, including conservation, codon usage, splice sites, splicing enhancers and suppressors, and mRNA folding free energy.	Buske et al., 2013
(7) Motif Break	It is an R/bioconductor software package that uses three algorithms for interrogation of genomes with motifs from many public sources. It judge the sequence surrounding polymorphism or mutation and how much information is gained or lost in one allele of the polymorphism or mutation, after it scores and reports the reference and alternate alleles of the sequence, and the effect (strong, weak or neutral) relative to the other.	Coetzee et al., 2015
(8) Human Splicing Finder (HSF)	Evaluates donor and acceptor splice sequences, branchpoint sequence and auxiliary sequences - such as exonic and intronic splicing enhancers and exonic and intronic splicing silencers - to predict possible splice changes	Desmet et al., 2009
(9) MethPrimer	MethPrimer, based on Primer3, is a program for designing PCR primers for methylation mapping. It first takes a DNA sequence as its input and searches the sequence for potential CpG islands.	Li and Dahiya, 2002
(10) miRBase	Predicts possible gains or losses of microRNAs (miRNAs) interactions by sequence complementarity	Griffiths-Jones et al., 2006

Supplementary Table 2. Linkage desequilibrium of variants in the same gene.

	ESCO2					
	rs35430328	rs2272730	rs4732748	rs182074852	rs62498042	rs114667641
rs35430328	-					
rs2272730	**1.0***	-				
rs4732748	1.0	1.0	-			
rs182074852	1.0	1.0	1.0	-		
rs62498042	**0.893***	**1.0***	0.122	1.0	-	
rs114667641	1.0	1.0	1.0	1.0	1.0	-

*High statistical significance (LOD>2)

Supplementary Table 2: Linkage desequilibrium of variants in the same gene (Continued).

	**SALL4**														
	rs150300174	rs3171177	rs138891224	rs17802735	rs6091375	rs13043248	rs6021437	rs6126344	rs140110863	rs13038893	rs61737139	rs6013281	rs146597375	rs190593855	rs11697572
rs150300174	-														
rs3171177	1.0	-													
rs138891224	1.0	1.0	-												
rs17802735	1.0	1.0	1.0	-											
rs6091375	1.0	1.0	1.0	0.04	-										
rs13043248	1.0	0.336	1.0	1.0	1.0	-									
rs6021437	1.0	1.0	1.0	1.0	1.0	1.0	-								
rs6126344	1.0	1.0	1.0	1.0	1.0	1.0	**1.0****	-							
rs140110863	1.0	1.0	1.0	1.0	1.0	1.0	1.0	1.0	-						
rs13038893	1.0	1.0	1.0	0.679	1.0	**1.0***	**1.0***	**1.0***	1.0	-					
rs61737139	1.0	**1.0***	1.0	1.0	1.0	0.336	1.0	1.0	1.0	1.0	-				
rs6013281	1.0	1.0	1.0	1.0	1.0	1.0	1.0	1.0	1.0	1.0	1.0	-			
rs146597375	1.0	1.0	1.0	1.0	1.0	1.0	1.0	1.0	1.0	1.0	1.0	1.0	-		
rs190593855	1.0	1.0	1.0	1.0	1.0	1.0	1.0	1.0	1.0	1.0	1.0	1.0	1.0	-	
rs11697572	1.0	1.0	1.0	0.153	**1.0***	1.0	1.0	1.0	1.0	1.0	1.0	1.0	1.0	1.0	-

*High statistical significance (LOD>2)

Supplementary Table 2: Linkage desequilibrium of variants in the same gene (Continued).

	TBX5														
	rs6489956	rs143511878	rs883079	rs28730761	rs147405081	rs78344365	rs2277377	rs2236017	rs185924249	c.420G>A	rs571924700	rs12423887	rs1248046	rs186960328	rs143563344
rs6489956	-														
rs143511878	1.0	-													
rs883079	**1.0**	1.0	-												
rs28730761	1.0	1.0	1.0	-											
rs147405081	0.106	1.0	1.0	1.0	-										
rs78344365	1.0	1.0	1.0	1.0	1.0	-									
rs2277377	1.0	1.0	**1.0**	1.0	0.038	1.0	-								
rs2236017	0.568	1.0	0.517	0.289	1.0	1.0	**1.0**	-							
rs185924249	1.0	1.0	1.0	1.0	1.0	1.0	1.0	1.0	-						
c.420G>A	1.0	1.0	1.0	1.0	1.0	1.0	1.0	1.0	1.0	-					
rs571924700	1.0	1.0	1.0	1.0	1.0	1.0	1.0	1.0	1.0	1.0	-				
rs12423887	1.0	1.0	0.341	0.273	1.0	1.0	1.0	1.0	1.0	1.0	1.0	-			
rs1248046	1.0	1.0	0.493	1.0	1.0	1.0	0.537	0.261	1.0	1.0	1.0	1.0	-		
rs186960328	0.106	1.0	1.0	1.0	**1.0**	1.0	0.038	1.0	1.0	1.0	1.0	1.0	1.0	-	
rs143563344	1.0	1.0	1.0	1.0	1.0	1.0	1.0	1.0	1.0	1.0	1.0	1.0	1.0	1.0	-

*High statistical significance (LOD>2)

Supplementary Table 3. Haplotypes of variants in the same gene.

**ESCO2**	**n**	**%**
GTCGAGA	23	0,43
ACCGAAA	12	0,22
GCCGAGA	7	0,13
GTCGGGA	4	0,07
GCTGAGA	2	0,04
ACCGAGA	2	0,04
others	4	0,07
**SALL4**	**n**	**%**
ACAAGCGATGTGCAA	17	0,31
ACAAGCACTGTGCAA	13	0,24
ACAAGTGATATGCAA	8	0,15
ACAGGCGATATGCAA	4	0,07
ACAACCGATATGCAG	3	0,06
ATAAGCGATAGGCAA	2	0,04
others	7	0,13
**TBX5**	**n**	**%**
ACTCGGGAGTCTCGC	9	0,17
GCTTGGGCTTCTCGC	7	0,13
GCTTGGGAGTCTCGC	6	0,11
GCCTTGAAGCTCCGC	5	0,09
GCCTTGAAGCTCTGC	3	0,06
GCTTTGAAGCTCCGC	3	0,06
ACCTTGACGCTCTGC	3	0,06
others	18	0,33

Supplementary Table 4. Funcional prediction of variants found in ESCO2, SALL4 and TBX5 genes.

Gene	Locus	Polymorphism	DNA variation	Position	Consequence	Aminoacid variation	SIFT	PolyPhen-2	MutationTaster	HSF	F-SNP
ESCO2	8:27631990	rs35430328	c.-151G>A	exon 1					Splicing affected; Protein features might be affected		Transcriptional regulation affected
	8:27632143	rs2272730	c.-17+19C>T	intron variant						ESE site broken	Transcriptional regulation affected
	8:27634064	rs4732748	c.239C>T	exon 3	missense	p.Ala80Val	tolerated	benign	Splicing affected; Protein features might be affected	New ESS site; ESE site broken	
	8:27634171	rs182074852	c.346G>A	exon 3	missense	p.Asp116Asn	tolerated	benign	Splicing affected; Protein features might be affected	ESE site broken	
	8:27641609	rs1824449	c.1013+35G>A	intron variant							
	8:27661025	rs533417099	c.*71_*74delTATT	3'UTR					Splicing affected		
	8:27661085	rs62498042	c.*130G>A	3'UTR					Splicing affected		
	8:27662444	rs114667641	c.*1489A>T	3'UTR					Splicing affected; Protein features might be affected		

Supplementary Table 4. Funcional prediction of variants found in ESCO2, SALL4 and TBX5 genes (Continued).

Gene	Locus	Polymorphism	DNA variation	Position	Consequence	Aminoacid variation	miRBase	MotifBreakR	MethPrimer	PredictSNP2	SILVA
ESCO2	8:27631990	rs35430328	c.-151G>A	exon 1			hsa-miR-6858		Changes 1 CpG island	Deleterious - 5 tools	
	8:27632143	rs2272730	c.-17+19C>T	intron variant			hsa-miR-6087	ZFX		Benign - 4 tools	
	8:27634064	rs4732748	c.239C>T	exon 3	missense	p.Ala80Val				Benign - 5 tools	
	8:27634171	rs182074852	c.346G>A	exon 3	missense	p.Asp116Asn				Benign - 4 tools	
	8:27641609	rs1824449	c.1013+35G>A	intron variant				TBP, POU6F1		Benign - 6 tools	
	8:27661025	rs533417099	c.*71_*74delTATT	3'UTR			hsa-miR-606, hsa-miR-3149				
	8:27661085	rs62498042	c.*130G>A	3'UTR						Benign - 5 tools	
	8:27662444	rs114667641	c.*1489A>T	3'UTR				ALX1, HOXA10		Benign - 6 tools	

Supplementary Table 4. Funcional prediction of variants found in ESCO2, SALL4 and TBX5 genes (Continued).

Gene	Locus	Polymorphism	DNA variation	Position	Consequence	Aminoacid variation	SIFT	PolyPhen-2	Mutation Taster	HSF	F-SNP
SALL4	20:50409207	rs11697572	c.131-316C>T	intron variant					Splicing affected; Protein features might be affected	ESE site broken	
	20:50409151	rs190593855	c.131-260G>A	intron variant					Splicing affected; Protein features might be affected	ESE site broken	
	20:50409117	rs146597375	c.131-226T>C	intron variant					Splicing affected; Protein features might be affected	ESE site broken	
	20:50408482	rs6013281	c.540T>C	exon 2	synonymous				Splicing affected; Protein features might be affected	New donor site; New ESS site; ESE site broken	Splicing affected
	20:50408377	rs61737139	c.645C>G	exon 2	synonymous						
	20:50407966	rs13038893	c.1056G>A	exon 2	synonymous				Splicing affected; Protein features might be affected		Splicing affected
	20:50407669	rs140110863	c.1353C>T	exon 2	synonymous						
	20:50407502	rs6126344	c.1520T>G	exon 2	missense	p.Leu507Arg	tolerated	benign	Splicing affected; Protein features might be affected		Splicing affected
	20:50407162	rs6021437	c.1860A>G	exon 2	synonymous				Splicing affected; Protein features might be affected		Splicing affected
	20:50406985	rs13043248	c.2037C>T	exon 2	synonymous				Splicing affected; Protein features might be affected	New ESS site	
	20:50406630	rs6091375	c.2392A>C	exon 2	missense	p.Ile798Leu	tolerated	benign	Splicing affected; Protein features might be affected	ESE site broken	Splicing affected
	20:50405502	rs17802735	c.2640G>C	exon 3	synonymous				Splicing affected; Protein features might be affected	New ESS site	Splicing affected
	20:50400989	rs138891224	c.2977G>C	exon 4	missense	p.Gly993Arg	damaging	possibly damaging	Disease causing; Splicing changed	ESE site broken	
	20:50400679	rs3171177	c.*125T>A	3'UTR						ESE site broken	Transcriptional regulation affected
	20:50400307	rs150300174	c.*497T>C	downstream						New ESS site	

Supplementary Table 4. Funcional prediction of variants found in ESCO2, SALL4 and TBX5 genes (Continued).

Gene	Locus	Polymorphism	DNA variation	Position	Consequence	Aminoacid variation	miRBase	MotifBreakR	MethPrimer	PredictSNP2	SILVA
SALL4	20:50409207	rs11697572	c.131-316C>T	intron variant			hsa-miR-1273c, hsa-miR-1285, hsa-miR-5096	PAX5		Benign - 6 tools	
	20:50409151	rs190593855	c.131-260G>A	intron variant			hsa-miR-222, hsa-miR-665, hsa-miR-4459, hsa-miR7851			Benign - 6 tools	
	20:50409117	rs146597375	c.131-226T>C	intron variant			hsa-miR-615	TFAP4, ZBTB7B		Benign - 5 tools	
	20:50408482	rs6013281	c.540T>C	exon 2	synonymous					Benign - 6 tools	Likely benign
	20:50408377	rs61737139	c.645C>G	exon 2	synonymous		hsa-miR-3911, hsa-miR-6766			Benign - 6 tools	Likely benign
	20:50407966	rs13038893	c.1056G>A	exon 2	synonymous				Lost of 1 CpG island	Benign - 6 tools	Likely benign
	20:50407669	rs140110863	c.1353C>T	exon 2	synonymous			MBD2		Benign - 6 tools	Likely benign
	20:50407502	rs6126344	c.1520T>G	exon 2	missense	p.Leu507Arg		NR2F2, PPARA, PPARD, PPARG, RARB, RXRA RXRB		Benign - 5 tools	
	20:50407162	rs6021437	c.1860A>G	exon 2	synonymous				Changes 2 CpG island	Benign - 6 tools	Likely benign
	20:50406985	rs13043248	c.2037C>T	exon 2	synonymous		hsa-miR-6874		Changes 1 CpG island	Benign - 6 tools	Likely benign
	20:50406630	rs6091375	c.2392A>C	exon 2	missense	p.Ile798Leu				Benign - 5 tools	
	20:50405502	rs17802735	c.2640G>C	exon 3	synonymous					Benign - 6 tools	Likely benign
	20:50400989	rs138891224	c.2977G>C	exon 4	missense	p.Gly993Arg				Deleterious - 4 tools	
	20:50400679	rs3171177	c.*125T>A	3'UTR						Benign - 4 tools	
	20:50400307	rs150300174	c.*497T>C	downstream			hsa-miR-5095, hsa-miR-1254, hsa-miR-5689	PITX2		Deleterious - 3 tools	

Supplementary Table 4. Funcional prediction of variants found in ESCO2, SALL4 and TBX5 genes (Continued).

Gene	Locus	Polymorphism	DNA variation	Position	Consequence	Aminoacid variation	SIFT	PolyPhen-2	Mutation Taster	HSF	F-SNP
TBX5	12:114846292	rs143563344	c.-712C>T	5'UTR							
	12:114846244	rs186960328	c.-664G>A	exon 1						New donor site; New ESS site; ESE site broken	
	12:114845506	rs1248046	c.-39+113A>G	intron variant					Splicing affected; Protein features might be affected		
	12:114843606	rs12423887	c.-38-1865G>A	intron variant					Splicing affected; Protein features might be affected	ESE site broken	
	12:114841450	rs571924700	c.147+107C>A	intron variant					Splicing affected; Protein features might be affected	New donor site	
	12:114836468	c.420G>A[b]	c.420C>T	exon 5	synonymous				Disease causing; Splicing changed; Protein features might be affected	ESE site broken	
	12:114832754	rs185924249	c.511-56T>C	intron variant					Disease causing; Splicing changed; Protein features might be affected		
	12:114832510	rs2236017	c.663+36G>T	intron variant					Splicing affected; Protein features might be affected		Transcriptional regulation affected
	12:114823187	rs2277377	c.755+94C>A	intron variant					Splicing affected; Protein features might be affected	ESE site broken	Transcriptional regulation affected
	12:114804222	rs78344365	c.756-26G>A	intron variant							
	12:114804165	rs147405081	c.787G>A	exon 8	missense	p.Val263Met	tolerated	possibly damaging	Disease causing; Splicing changed; Protein features might be affected	New ESS site	
	12:114793260	rs28730761	c.*77A>G	3'UTR					Splicing affected		
	12:114793240	rs883079	c.*97G>A	3'UTR						New ESS site	
	12:114792525	rs143511878	c.*812C>T	3'UTR					Splicing affected	ESE site broken	
	12:114792236	rs6489956	c.*1101A>G	3'UTR					Splicing affected		

Supplementary Table 4. Funcional prediction of variants found in ESCO2, SALL4 and TBX5 genes (Continued).

Gene	Locus	Polymorphism	DNA variation	Position	Consequence	ninoacid variati	miRBase	MotifBreakR	MethPrimer	PredictSNP2	SILVA
TBX5	12:114846292	rs143563344	c.-712C>T	5'UTR						Deleterious - 4 tools	
	12:114846244	rs186960328	c.-664G>A	exon 1			hsa-miR-6763	POU6F1		Benign - 3 tools	
	12:114845506	rs1248046	c.-39+113A>G	intron variant						Benign - 5 tools	
	12:114843606	rs12423887	c.-38-1865G>A	intron variant				NFIB	Changes 1 CpG island	Deleterious - 6 tools	
	12:114841450	rs571924700	c.147+107C>A	intron variant					Changes 1 CpG island	Benign - 5 tools	
	12:114836468	c.420G>A[b]	c.420C>T	exon 5	synonymous					Benign - 5 tools	Likely benign
	12:114832754	rs185924249	c.511-56T>C	intron variant				GATA1, GATA2		Deleterious - 4 tools	
	12:114832510	rs2236017	c.663+36G>T	intron variant						Benign - 5 tools	
	12:114823187	rs2277377	c.755+94C>A	intron variant			hsa-miR-1207, hsa-miR-6893			Benign - 4 tools	
	12:114804222	rs78344365	c.756-26G>A	intron variant						Benign - 6 tools	
	12:114804165	rs147405081	c.787G>A	exon 8	missense	p.Val263Met				Deleterious - 4 tools	
	12:114793260	rs28730761	c.*77A>G	3'UTR			hsa-miR-6876, hsa-miR-329, hsa-miR-6510			Deleterious - 5 tools	
	12:114793240	rs883079	c.*97G>A	3'UTR			hsa-miR-492, hsa-miR-6510			Benign - 4 tools	
	12:114792525	rs143511878	c.*812C>T	3'UTR				ELF1		Benign - 4 tools	
	12:114792236	rs6489956	c.*1101A>G	3'UTR						Benign - 5 tools	

Supplementary Table 5. Scores.

Gene	Polymorphism	DNA variation	SIFT	PolyPhen-2	Mutation Taster	HSF	F-SNP	miRBase	Motif Break R	MethPrimer	PredictSNP2	SILVA	Haploview	Chi-Square	Score Final
ESCO2	rs35430328	c.-151G>A			1+1		1	1		1	1+1				7
	rs2272730	c.-17+19C>T				1	1	1	1						4
	rs4732748	c.239C>T	0	0	1+1	1+1									4
	rs182074852	c.346G>A	0	0	1+1	1									3
	rs1824449	c.1013+35G>A							1+1					1+1+2	6
	rs533417099	c.*71_*74delTATT			1			1						1+2	5
	rs62498042	c.*130G>A			1									1	2
	rs114667641	c.*1489A>T			1+1				2						4

Supplementary Table 5. Scores (Continued).

Gene	Polymorphism	DNA variation	SIFT	PolyPhen-2	Mutation Taster	HSF	F-SNP	miRBase	Motif Break R	MethPrimer	PredictSNP2	SILVA	Haploview	Chi-Square	Score Final
SALL4	rs11697572	c.131-316C>T			1+1	1		1	1						5
	rs190593855	c.131-260G>A			1+1	1		1							4
	rs146597375	c.131-226T>C			1+1	1		1	2						6
	rs6013281	c.540T>C			1+1	1+1+1	1					0			6
	rs61737139	c.645C>G						1				0			1
	rs13038893	c.1056G>A			1+1		1			3		0			6
	rs140110863	c.1353C>T							1			0		1	2
	rs6126344	c.1520T>G	0	0	1+1		1		7				1		11
	rs6021437	c.1860A>G			1+1		1			1+1		0	1		6
	rs13043248	c.2037C>T			1+1	1		1		1		0			5
	rs6091375	c.2392A>C	0	0	1+1	1	1								4
	rs17802735	c.2640G>C			1+1	1	1					0			4
	rs138891224	c.2977G>C	3	3	1+3	1					1+1			1+1	15
	rs3171177	c.*125T>A				1	1								2
	rs150300174	c.*497T>C				1		2	1		1+1			1	7

Supplementary Table 5. Scores (Continued).

Gene	Polymorphism	DNA variation	SIFT	PolyPhen-2	Mutation Taster	HSF	F-SNP	miRBase	Motif Break R	MethPrimer	PredictSNP2	SILVA	Haploview	Chi-Square	Score Final
	rs143563344	c.-712C>T									1+1				2
	rs186960328	c.-664G>A				1+1+1		1	1						5
	rs1248046	c.-39+113A>G			1+1										2
	rs12423887	c.-38-1865G>A			1+1	1			1	1	1+1				8
	rs571924700	c.147+107C>A			1+1	1				1				1	4
	c.420G>A	c.420C>T			1+1+3	1									6
	rs185924249	c.511-56T>C			1+1+3				2		1				8
TBX5	rs2236017	c.663+36G>T			1+1		1								3
	rs2277377	c.755+94C>A			1+1	1	1	1							5
	rs78344365	c.756-26G>A												1	1
	rs147405081	c.787G>A	0	3	1+1+3	1					1			1+1+2	14
	rs28730761	c.*77A>G			1			1			1+1				4
	rs883079	c.*97G>A				1		1						1	3
	rs143511878	c.*812C>T			1	1			1						3
	rs6489956	c.*1101A>G			1										1

Legends and scores (supplementary table 5)

Tool	Changes	Score	Obs
**SIFT/Polyphen-2/SILVA**	Tolerated, likely benign or neutral	0	
	Possibly Damaging or damaging	+3	per tool
**Mutation Taster**	Polymorphism	0	
	Splicing	+1	
	Protein features might be affected	+1	
	Disease causing	+3	
**HSF**	No alteration of splicing	0	
	Alteration of ESSE/ESS	+1	per site
	Activation exonic cryptic acceptor/donor site	+1	per site
**F-SNP**	Splicing	+1	
	transcriptional regulation affected	+1	
**miRBase**	non-affected miRNAs	0	
	unrelated miRNA affected	+1	
	related miRNA affected	+2	per site
**Motif Break**	Does not affect TF	0	
	Affects TF with ontology for limbs or heart development	+1	per TF
	Affects TF with binding site on the gene	+1	per TF
**MethPrimer**	Does not change island	0	
	Change start or end of CpG island	+1	por island
	Break Island	+3	por island
**Predict SNP**	Pathogenic	+2	
	Pathogenic included DANN	+1	
**Haploview**	Block	+1	
**Fisher/Chi-Square**	p<0.05 1000 Genomes	+1	
	p<0.05 ExAc	+1	
	p<0.05 ABraOM	+2	

Supplementary Table 6. Gene Ontology.

#pathway ID	pathway description	observed gene count	false discovery rate	matching proteins in your network (labels)
GO.0051276	chromosome organization	18	3.71e-14	CCNB1,DNMT3B,ESPL1,HDAC1,HDAC2,HDAC4,MAD2L1,NCAPG,PCNA,PDS5A,PDS5B,POLA2,RAD21L1,RFC3,RFC4,RFC5,SMARCA2,SMARCA4
GO.0000278	mitotic cell cycle	16	8.36e-12	CCNB1,CCNB2,CDC7,ESCO2,ESPL1,HDAC1,NCAPG,PCNA,PDS5A,PDS5B,POLA2,RAD21,RFC3,RFC4,RFC5,SMARCA4
GO.0007049	cell cycle	17	3.65e-10	CCNB1,CCNB2,CDC7,CHTF18,ESCO2,HDAC1,NCAPG,PCNA,PDS5A,PDS5B,POLA2,RAD21,RAD21L1,RFC3,RFC4,RFC5,SMARCA4
GO.1902589	single-organism organelle organization	19	5.2e-10	CCNB1,CCNB2,DNMT3B,HDAC1,HDAC2,HDAC4,MAD2L1,NCAPG,NKX2-5,PCNA,PDS5A,PDS5B,POLA2,RAD21,RAD21L1,RFC3,RFC4,RFC5,SMARCA4
GO.0098813	nuclear chromosome segregation	8	1.31e-08	CCNB1,MAD2L1,NCAPG,PDS5A,PDS5B,RAD21L1,REC8,SMC3
GO.0000070	mitotic sister chromatid segregation	7	1.69e-08	CCNB1,ESPL1,MAD2L1,NCAPG,PDS5A,PDS5B,SMC3
GO.0006996	organelle organization	20	1.69e-08	CCNB1,CCNB2,DNMT3B,HDAC1,HDAC2,HDAC4,MAD2L1,NCAPG,NKX2-5,PCNA,PDS5A,PDS5B,POLA2,RAD21,RAD21L1,RFC3,RFC4,RFC5,SMARCA2,SMARCA4
GO.0022402	cell cycle process	14	2.8e-08	CCNB1,CCNB2,CDC7,NCAPG,PCNA,PDS5A,PDS5B,POLA2,RAD21,RAD21L1,RFC3,RFC4,RFC5,SMARCA4
GO.0032201	telomere maintenance via semi-conservative replication	5	1.32e-07	PCNA,POLA2,RFC3,RFC4,RFC5
GO.0006281	DNA repair	10	1.91e-07	CDC7,ESCO2,PCNA,RAD21,RAD21L1,REC8,RFC3,RFC4,RFC5,SMC3
GO.0007059	chromosome segregation	8	1.91e-07	CCNB1,ESCO2,MAD2L1,NCAPG,PDS5A,PDS5B,RAD21,RAD21L1
GO.0006310	DNA recombination	8	2.19e-07	CDC7,PCNA,POLA2,RAD21,RAD21L1,RFC3,RFC4,RFC5
GO.0000722	telomere maintenance via recombination	5	2.29e-07	PCNA,POLA2,RFC3,RFC4,RFC5
GO.0033260	nuclear DNA replication	5	2.29e-07	PCNA,POLA2,RFC3,RFC4,RFC5
GO.0051301	cell division	10	3.86e-07	CCNB1,CCNB2,CDC7,ESPL1,MAD2L1,NCAPG,PDS5A,PDS5B,RAD21,SMC3
GO.0007067	mitotic nuclear division	9	4.82e-07	CCNB1,CCNB2,ESPL1,MAD2L1,NCAPG,PDS5A,PDS5B,RAD21,SMARCA4
GO.0006259	DNA metabolic process	11	6.14e-07	CDC7,CHTF18,ESCO2,PCNA,POLA2,RAD21,RAD21L1,RFC3,RFC4,RFC5,SMC3
GO.0006271	DNA strand elongation involved in DNA replication	5	6.14e-07	PCNA,POLA2,RFC3,RFC4,RFC5
GO.1903047	mitotic cell cycle process	11	1.02e-06	CCNB1,CCNB2,CDC7,ESPL1,NCAPG,PCNA,PDS5A,PDS5B,POLA2,RAD21,SMARCA4
GO.0006261	DNA-dependent DNA replication	6	1.21e-06	CDC7,PCNA,POLA2,RFC3,RFC4,RFC5
GO.0000280	nuclear division	9	2.34e-06	CCNB1,CCNB2,MAD2L1,NCAPG,PDS5A,PDS5B,RAD21,RAD21L1,SMARCA4
GO.0006260	DNA replication	7	5.35e-06	CDC7,CHTF18,PCNA,POLA2,RFC3,RFC4,RFC5
GO.0006297	nucleotide-excision repair, DNA gap filling	4	5.35e-06	PCNA,RFC3,RFC4,RFC5
GO.0070987	error-free translesion synthesis	4	5.35e-06	PCNA,RFC3,RFC4,RFC5

Supplementary Table 6. Gene Ontology (Continued).

GO.0042276	error-prone translesion synthesis	4	6.35e-06	PCNA,RFC3,RFC4,RFC5
GO.0033554	cellular response to stress	13	2.33e-05	CCNB1,CDC7,DNMT3B,ESCO2,HDAC2,PCNA,RAD21,RAD21L1,REC8,RFC3,RFC4,RFC5,SMC3
GO.0045935	positive regulation of nucleobase-containing compound metabolic process	13	2.81e-05	CCNB1,CDC7,HDAC1,HDAC2,HDAC4,NANOG,NKX2-5,PCNA,RAD21,SALL4,SMARCA2,SMARCA4,TBX5
GO.0071897	DNA biosynthetic process	5	4.41e-05	PCNA,POLA2,RFC3,RFC4,RFC5
GO.0042769	DNA damage response, detection of DNA damage	4	5.68e-05	PCNA,RFC3,RFC4,RFC5
GO.0070933	histone H4 deacetylation	3	0.000111	HDAC1,HDAC2,HDAC4
GO.0010557	positive regulation of macromolecule biosynthetic process	12	0.000142	CDC7,HDAC1,HDAC2,HDAC4,NANOG,NKX2-5,PCNA,RAD21,SALL4,SMARCA2,SMARCA4,TBX5
GO.0045944	positive regulation of transcription from RNA polymerase II promoter	10	0.000142	HDAC1,HDAC2,HDAC4,NANOG,NKX2-5,RAD21,SALL4,SMARCA2,SMARCA4,TBX5
GO.0006283	transcription-coupled nucleotide-excision repair	4	0.00018	PCNA,RFC3,RFC4,RFC5
GO.0007064	mitotic sister chromatid cohesion	3	0.000186	PDS5A,PDS5B,SMC3
GO.0006275	regulation of DNA replication	5	0.000193	CDC7,ESCO2,PCNA,PDS5A,SMC3
GO.0006284	base-excision repair	4	0.000193	PCNA,RFC3,RFC4,RFC5
GO.0006357	regulation of transcription from RNA polymerase II promoter	12	0.000193	DNMT3B,HDAC1,HDAC2,HDAC4,NANOG,NKX2-5,PCNA,RAD21,SALL4,SMARCA2,SMARCA4,TBX5
GO.0010628	positive regulation of gene expression	12	0.000193	CCNB1,DNMT3B,HDAC1,HDAC2,HDAC4,NANOG,NKX2-5,RAD21,SALL4,SMARCA2,SMARCA4,TBX5
GO.0031328	positive regulation of cellular biosynthetic process	12	0.000249	CDC7,HDAC1,HDAC2,HDAC4,NANOG,NKX2-5,PCNA,RAD21,SALL4,SMARCA2,SMARCA4,TBX5
GO.0006302	double-strand break repair	5	0.000313	CDC7,ESCO2,RAD21,RAD21L1,REC8
GO.0051254	positive regulation of RNA metabolic process	11	0.000418	CCNB1,HDAC1,HDAC2,HDAC4,NANOG,NKX2-5,RAD21,SALL4,SMARCA2,SMARCA4,TBX5
GO.0090304	nucleic acid metabolic process	17	0.00044	CDC7,CHTF18,ESCO2,HDAC1,HDAC2,HDAC4,NANOG,PCNA,POLA2,RAD21L1,RFC3,RFC4,RFC5,SALL4,SMARCA2,SMARCA4,SMC3
GO.0010604	positive regulation of macromolecule metabolic process	14	0.000476	CDC7,DNMT3B,ESPL1,HDAC1,HDAC2,HDAC4,NANOG,NKX2-5,PCNA,RAD21,SALL4,SMARCA2,SMARCA4,TBX5
GO.0006338	chromatin remodeling	5	0.000628	HDAC1,HDAC2,HDAC4,SMARCA2,SMARCA4
GO.0051726	regulation of cell cycle	9	0.000685	CCNB1,CCNB2,CDC7,ESPL1,HDAC1,HDAC2,MAD2L1,SMARCA4,SMC3
GO.0070932	histone H3 deacetylation	3	0.000841	HDAC1,HDAC2,HDAC4
GO.0019219	regulation of nucleobase-containing compound metabolic process	16	0.000853	CCNB1,CDC7,ESCO2,HDAC1,HDAC2,HDAC4,NANOG,NKX2-5,PCNA,PDS5A,RAD21,SALL4,SMARCA2,SMARCA4,SMC3,TBX5
GO.0031325	positive regulation of cellular metabolic process	14	0.000951	CDC7,DNMT3B,ESPL1,HDAC1,HDAC2,HDAC4,NANOG,NKX2-5,PCNA,RAD21,SALL4,SMARCA2,SMARCA4,TBX5
GO.0045934	negative regulation of nucleobase-containing compound metabolic process	10	0.00106	HDAC1,HDAC2,HDAC4,NANOG,NKX2-5,PDS5A,SALL4,SMARCA2,SMARCA4,SMC3
GO.0010558	negative regulation of macromolecule biosynthetic process	10	0.00116	HDAC1,HDAC2,HDAC4,NANOG,NKX2-5,PDS5A,SALL4,SMARCA2,SMARCA4,SMC3

Supplementary Table 6. Gene Ontology (Continued).

GO.0000122	negative regulation of transcription from RNA polymerase II promoter	8	0.0012	DNMT3B,HDAC1,HDAC2,HDAC4,NKX2-5,SALL4,SMARCA2,SMARCA4
GO.0016568	chromatin modification	7	0.00126	CCNB1,DNMT3B,HDAC1,HDAC2,HDAC4,SMARCA2,SMARCA4
GO.0061198	fungiform papilla formation	2	0.00126	HDAC1,HDAC2
GO.0031327	negative regulation of cellular biosynthetic process	10	0.00163	HDAC1,HDAC2,HDAC4,NANOG,NKX2-5,PDS5A,SALL4,SMARCA2,SMARCA4,SMC3
GO.0007062	sister chromatid cohesion	3	0.00172	PDS5A,PDS5B,REC8
GO.0003166	bundle of His development	2	0.00217	NKX2-5,TBX5
GO.0010605	negative regulation of macromolecule metabolic process	12	0.00222	CCNB1,HDAC1,HDAC2,HDAC4,MAD2L1,NANOG,NKX2-5,PDS5A,SALL4,SMARCA2,SMARCA4,SMC3
GO.0030154	cell differentiation	14	0.00222	CCNB1,DNMT3B,ESCO2,HDAC1,HDAC2,NANOG,NKX2-5,PCNA,REC8,SALL4,SMARCA2,SMARCA4,SMC3,TBX5
GO.0031324	negative regulation of cellular metabolic process	12	0.00235	CCNB1,HDAC1,HDAC2,HDAC4,MAD2L1,NANOG,NKX2-5,PDS5A,SALL4,SMARCA2,SMARCA4,SMC3
GO.0048513	organ development	13	0.00235	CCNB1,CCNB2,ESCO2,HDAC1,HDAC2,HDAC4,NKX2-5,PCNA,RAD21L1,REC8,SALL4,SMARCA4,TBX5
GO.2000112	regulation of cellular macromolecule biosynthetic process	15	0.00275	CDC7,ESCO2,HDAC1,HDAC2,HDAC4,NANOG,NKX2-5,PCNA,PDS5A,RAD21,SALL4,SMARCA2,SMARCA4,SMC3,TBX5
GO.0045143	homologous chromosome segregation	3	0.00306	ESPL1,RAD21L1,REC8
GO.0045931	positive regulation of mitotic cell cycle	4	0.00306	CCNB1,CDC7,ESPL1,MAD2L1
GO.0003164	His-Purkinje system development	2	0.00307	NKX2-5,TBX5
GO.0051891	positive regulation of cardioblast differentiation	2	0.00307	NKX2-5,TBX5
GO.0060789	hair follicle placode formation	2	0.00307	HDAC1,HDAC2
GO.0061197	fungiform papilla morphogenesis	2	0.00307	HDAC1,HDAC2
GO.0034645	cellular macromolecule biosynthetic process	15	0.00319	CDC7,CHTF18,HDAC1,HDAC2,HDAC4,NANOG,PCNA,POLA2,RAD21,RFC3,RFC4,RFC5,SALL4,SMARCA2,SMARCA4
GO.0045596	negative regulation of cell differentiation	7	0.0034	HDAC2,HDAC4,NANOG,NKX2-5,SALL4,SMC3,TBX5
GO.1901990	regulation of mitotic cell cycle phase transition	5	0.00343	CCNB1,CDC7,ESPL1,MAD2L1,SMARCA4
GO.0048468	cell development	10	0.00386	CCNB1,HDAC2,HDAC4,NANOG,NKX2-5,REC8,SALL4,SMARCA2,SMARCA4,SMC3
GO.0009790	embryo development	8	0.00388	CCNB1,CCNB2,HDAC1,HDAC2,NANOG,NKX2-5,SALL4,TBX5
GO.0045786	negative regulation of cell cycle	6	0.00397	ESPL1,HDAC1,HDAC2,MAD2L1,SMARCA4,SMC3
GO.0061196	fungiform papilla development	2	0.004	HDAC1,HDAC2
GO.2000113	negative regulation of cellular macromolecule biosynthetic process	9	0.004	HDAC1,HDAC2,HDAC4,NKX2-5,PDS5A,SALL4,SMARCA2,SMARCA4,SMC3
GO.0030326	embryonic limb morphogenesis	4	0.00407	HDAC1,HDAC2,SALL4,TBX5
GO.0003281	ventricular septum development	3	0.00442	NKX2-5,SALL4,TBX5

Supplementary Table 6. Gene Ontology (Continued).

GO.0006950	response to stress	14	0.00474	CCNB1,CDC7,DNMT3B,ESCO2,HDAC1,HDAC4,PCNA,RAD21,RAD21L1,REC8,RFC3,RFC4,RFC5,SMC3
GO.0044763	single-organism cellular process	24	0.00474	CCNB1,CCNB2,CDC7,CHTF18,DNMT3B,HDAC1,HDAC2,HDAC4,NANOG,NCAPG,NKX2-5,PCNA,PDS5A,PDS5B,POLA2,RAD21,RAD21L1,RFC3,RFC4,RFC5,SALL4,SMARCA2,SMARCA4,TBX5
GO.0072520	seminiferous tubule development	2	0.00495	RAD21L1,REC8
GO.2000738	positive regulation of stem cell differentiation	3	0.00528	HDAC2,NKX2-5,TBX5
GO.0051128	regulation of cellular component organization	11	0.00616	CCNB1,CDC7,DNMT3B,ESPL1,HDAC2,HDAC4,MAD2L1,NKX2-5,SMARCA2,SMARCA4,TBX5
GO.0048731	system development	14	0.0066	CCNB1,CCNB2,DNMT3B,ESCO2,HDAC1,HDAC2,NKX2-5,PCNA,RAD21L1,REC8,SALL4,SMARCA2,SMARCA4,TBX5
GO.0007275	multicellular organismal development	15	0.00681	CCNB1,CCNB2,DNMT3B,ESCO2,HDAC1,HDAC2,NANOG,NKX2-5,PCNA,RAD21L1,REC8,SALL4,SMARCA2,SMARCA4,TBX5
GO.0010564	regulation of cell cycle process	6	0.00746	CCNB1,CDC7,ESPL1,MAD2L1,SMARCA4,SMC3
GO.0044772	mitotic cell cycle phase transition	5	0.00746	CCNB1,CCNB2,CDC7,PCNA,POLA2
GO.0008284	positive regulation of cell proliferation	7	0.00805	CCNB1,CDC7,HDAC1,HDAC2,HDAC4,NKX2-5,TBX5
GO.0060255	regulation of macromolecule metabolic process	17	0.00805	CDC7,ESCO2,ESPL1,HDAC1,HDAC2,HDAC4,MAD2L1,NANOG,NKX2-5,PCNA,PDS5A,RAD21,SALL4,SMARCA2,SMARCA4,SMC3,TBX5
GO.0016569	covalent chromatin modification	5	0.00807	CCNB1,DNMT3B,HDAC1,HDAC2,HDAC4
GO.0034654	nucleobase-containing compound biosynthetic process	13	0.0082	HDAC1,HDAC2,HDAC4,NANOG,PCNA,POLA2,RAD21,RFC3,RFC4,RFC5,SALL4,SMARCA2,SMARCA4
GO.0044710	single-organism metabolic process	15	0.00822	CCNB1,CDC7,DNMT3B,ESCO2,HDAC1,HDAC2,HDAC4,PCNA,POLA2,RAD21,RAD21L1,RFC3,RFC4,RFC5,SMC3
GO.0060173	limb development	4	0.00865	HDAC1,HDAC2,SALL4,TBX5
GO.0080090	regulation of primary metabolic process	17	0.00865	CDC7,ESCO2,ESPL1,HDAC1,HDAC2,HDAC4,MAD2L1,NANOG,NKX2-5,PCNA,PDS5A,RAD21,SALL4,SMARCA2,SMARCA4,SMC3,TBX5
GO.0003211	cardiac ventricle formation	2	0.00896	NKX2-5,TBX5
GO.0043587	tongue morphogenesis	2	0.00896	HDAC1,HDAC2
GO.0051129	negative regulation of cellular component organization	6	0.00915	CCNB1,DNMT3B,ESPL1,HDAC2,MAD2L1,TBX5
GO.1903507	negative regulation of nucleic acid-templated transcription	8	0.00943	HDAC1,HDAC2,HDAC4,NANOG,NKX2-5,SALL4,SMARCA2,SMARCA4
GO.0048598	embryonic morphogenesis	6	0.00944	HDAC1,HDAC2,NANOG,NKX2-5,SALL4,TBX5
GO.0000082	G1/S transition of mitotic cell cycle	4	0.00958	CCNB1,CDC7,PCNA,POLA2
GO.0051983	regulation of chromosome segregation	3	0.0101	CCNB1,ESPL1,MAD2L1
GO.0061029	eyelid development in camera-type eye	2	0.0101	HDAC1,HDAC2
GO.0071168	protein localization to chromatin	2	0.0101	ESCO2,RAD21
GO.0007127	meiosis I	3	0.0103	ESPL1,RAD21,RAD21L1

Supplementary Table 6. Gene Ontology (Continued).

GO.0035295	tube development	6	0.0104	CCNB1,NKX2-5,RAD21L1,REC8,SALL4,TBX5
GO.0048522	positive regulation of cellular process	15	0.0111	CCNB1,CDC7,ESPL1,HDAC1,HDAC2,HDAC4,MAD2L1,NANOG,NKX2-5,PCNA,RAD21,SALL4,SMARCA2,SMARCA4,TBX5
GO.0010870	positive regulation of receptor biosynthetic process	2	0.0114	HDAC1,HDAC2
GO.0055015	ventricular cardiac muscle cell development	2	0.0114	CCNB1,NKX2-5
GO.0090266	regulation of mitotic cell cycle spindle assembly checkpoint	2	0.0114	CCNB1,MAD2L1
GO.0031323	regulation of cellular metabolic process	17	0.0117	CDC7,ESCO2,ESPL1,HDAC1,HDAC2,HDAC4,MAD2L1,NANOG,NKX2-5,PCNA,PDS5A,RAD21,SALL4,SMARCA2,SMARCA4,SMC3,TBX5
GO.0043044	ATP-dependent chromatin remodeling	3	0.0129	HDAC1,HDAC2,SMARCA4
GO.0060413	atrial septum morphogenesis	2	0.0129	NKX2-5,TBX5
GO.0060766	negative regulation of androgen receptor signaling pathway	2	0.0129	HDAC1,SMARCA4
GO.0000724	double-strand break repair via homologous recombination	3	0.0132	CDC7,RAD21L1,REC8
GO.0048523	negative regulation of cellular process	14	0.0142	CCNB1,ESPL1,HDAC1,HDAC2,HDAC4,MAD2L1,NANOG,NKX2-5,PDS5A,PDS5B,SMARCA2,SMARCA4,SMC3,TBX5
GO.0055007	cardiac muscle cell differentiation	3	0.0142	CCNB1,NKX2-5,TBX5
GO.0031000	response to caffeine	2	0.0144	DNMT3B,HDAC2
GO.0071696	ectodermal placode development	2	0.0144	HDAC1,HDAC2
GO.0071697	ectodermal placode morphogenesis	2	0.0144	HDAC1,HDAC2
GO.0033044	regulation of chromosome organization	4	0.0147	CCNB1,DNMT3B,ESPL1,MAD2L1
GO.0007281	germ cell development	4	0.0152	CCNB1,REC8,SMARCA2,SMARCA4
GO.0010832	negative regulation of myotube differentiation	2	0.0162	HDAC4,NKX2-5
GO.0051984	positive regulation of chromosome segregation	2	0.0162	CCNB1,ESPL1
GO.0050793	regulation of developmental process	10	0.018	CCNB1,CDC7,DNMT3B,HDAC2,HDAC4,NANOG,NKX2-5,SALL4,SMC3,TBX5
GO.0003283	atrial septum development	2	0.0182	NKX2-5,TBX5
GO.0007130	synaptonemal complex assembly	2	0.0182	RAD21L1,REC8
GO.0048856	anatomical structure development	14	0.0219	CCNB1,CCNB2,DNMT3B,ESCO2,HDAC1,HDAC2,NANOG,NKX2-5,RAD21L1,REC8,SMARCA2,SMARCA4,SMC3,TBX5
GO.2001252	positive regulation of chromosome organization	3	0.0221	CCNB1,DNMT3B,ESPL1
GO.0001556	oocyte maturation	2	0.0225	CCNB1,REC8
GO.0010948	negative regulation of cell cycle process	4	0.0231	ESPL1,MAD2L1,SMARCA4,SMC3
GO.2001251	negative regulation of chromosome organization	3	0.0233	DNMT3B,ESPL1,MAD2L1
GO.0007507	heart development	5	0.0238	CCNB1,NKX2-5,PCNA,SALL4,TBX5

Supplementary Table 6. Gene Ontology (Continued).

GO.0060045	positive regulation of cardiac muscle cell proliferation	2	0.0243	CCNB1,TBX5
GO.0003231	cardiac ventricle development	3	0.0256	NKX2-5,SALL4,TBX5
GO.0090068	positive regulation of cell cycle process	4	0.0261	CCNB1,CDC7,ESPL1,MAD2L1
GO.0003300	cardiac muscle hypertrophy	2	0.0274	HDAC2,HDAC4
GO.0016202	regulation of striated muscle tissue development	3	0.0274	CCNB1,HDAC4,NKX2-5
GO.0042692	muscle cell differentiation	4	0.0274	CCNB1,NKX2-5,SMARCA2,TBX5
GO.0043586	tongue development	2	0.0274	HDAC1,HDAC2
GO.0043921	modulation by host of viral transcription	2	0.0274	HDAC1,SMARCA4
GO.0044707	single-multicellular organism process	16	0.0274	CCNB1,CCNB2,DNMT3B,ESCO2,HDAC1,HDAC2,HDAC4,NANOG,NKX2-5,PCNA,RAD21L1,REC8,SMARCA2,SMARCA4,SMC3,TBX5
GO.0055012	ventricular cardiac muscle cell differentiation	2	0.0274	CCNB1,NKX2-5
GO.0065007	biological regulation	22	0.0274	CCNB1,CDC7,ESCO2,ESPL1,HDAC1,HDAC2,HDAC4,MAD2L1,NANOG,NKX2-5,PCNA,PDS5A,PDS5B,POLA2,RAD21,RFC3,RFC4,RFC5,SMARCA2,SMARCA4,SMC3,TBX5
GO.0010629	negative regulation of gene expression	8	0.0281	CCNB1,HDAC1,HDAC2,HDAC4,NKX2-5,SALL4,SMARCA2,SMARCA4
GO.0007126	meiotic nuclear division	3	0.0324	RAD21,RAD21L1,SMC3
GO.0042127	regulation of cell proliferation	8	0.0331	CCNB1,CDC7,HDAC1,HDAC2,HDAC4,NKX2-5,PDS5B,SMARCA2
GO.0045595	regulation of cell differentiation	8	0.0331	DNMT3B,HDAC2,HDAC4,NANOG,NKX2-5,SALL4,SMC3,TBX5
GO.0007286	spermatid development	3	0.0361	REC8,SMARCA2,SMARCA4
GO.0030261	chromosome condensation	2	0.0361	CCNB1,NCAPG
GO.0045892	negative regulation of transcription, DNA-templated	7	0.0361	HDAC1,HDAC2,HDAC4,NKX2-5,SALL4,SMARCA2,SMARCA4
GO.0072358	cardiovascular system development	6	0.0361	CCNB1,NKX2-5,PCNA,SALL4,SMARCA2,TBX5
GO.0072359	circulatory system development	6	0.0361	CCNB1,NKX2-5,PCNA,SALL4,SMARCA2,TBX5
GO.0010639	negative regulation of organelle organization	4	0.0382	CCNB1,DNMT3B,ESPL1,MAD2L1
GO.0048515	spermatid differentiation	3	0.0399	REC8,SMARCA2,SMARCA4
GO.0003209	cardiac atrium morphogenesis	2	0.0406	NKX2-5,TBX5
GO.0006270	DNA replication initiation	2	0.0406	CDC7,POLA2
GO.0009888	tissue development	8	0.0434	CCNB1,HDAC1,HDAC2,NANOG,NKX2-5,PCNA,SALL4,SMARCA4
GO.0060043	regulation of cardiac muscle cell proliferation	2	0.0464	CCNB1,NKX2-5
GO.0003230	cardiac atrium development	2	0.0484	NKX2-5,TBX5
GO.0051252	regulation of RNA metabolic process	12	0.0484	CCNB1,HDAC1,HDAC2,HDAC4,NANOG,NKX2-5,PCNA,RAD21,SALL4,SMARCA2,SMARCA4,TBX5
GO.0055093	response to hyperoxia	2	0.0484	DNMT3B,HDAC2

Supplementary Table 7. SNPs in cardiac diseases.

Polymorphism	DNA variation	Type	Gene	Disease	Reference
rs13038893	c.1056G>A	synonymous variant	*SALL4*	ventricular septal defects	Wang et al., 2009
rs6021437	c.1860A>G	synonymous variant	*SALL4*	ventricular septal defects	Wang et al., 2010
rs13043248	c.2037C>T	synonymous variants	*SALL4*	ventricular septal defects	Wang et al., 2011
rs17802735	c.2640G>C	synonymous variants	*SALL4*	ventricular septal defects	Wang et al., 2012
rs6126344	c.1520T>G	missense variants	*SALL4*	ventricular septal defects	Wang et al., 2013
rs6091375	c.2392A>C	missense variants	*SALL4*	ventricular septal defects	Wang et al., 2014
rs147405081	c.787G>A	missense variants	*TBX5*	bicuspid aortic valve	Bonachea et al., 2014
rs883079	c.*97G>A	3'UTR	*TBX5*	ventricular depolarization	Pazoki et al., 2013
rs6489956	c.*1101A>G	3'UTR	*TBX5*	congenital heart disease	Wang et al., 2017

Reference sequences are: ESCO2 transcript NM_001017420, SALL4 transcript NM_020436 and TBX5 transcript NM_000192.
